# S198. PRE-ADOLESCENT BRAIN STRUCTURE: THE INTERPLAY BETWEEN GENETIC VULNERABILITY FOR SCHIZOPHRENIA AND CORTISOL LEVELS

**DOI:** 10.1093/schbul/sby018.985

**Published:** 2018-04-01

**Authors:** Koen Bolhuis, Philip R Jansen, Hanan El Marroun, Manon H J Hillegers, Henning Tiemeier, Steven A Kushner

**Affiliations:** 1 Erasmus Medical Center; 2 Erasmus Medical Center, Vrije Universiteit Amsterdam; 3 Erasmus Medical Center, University Medical Center Utrecht

## Abstract

**Background:**

Schizophrenia is a highly heritable disease, mediated through a combination of common and rare genetic variants. In addition to genetic risk, several putative environmental risk factors have been studied in the context of schizophrenia. It has been suggested that the genetic effects on the etiology of schizophrenia may be of limited explanatory power if not viewed in the context of interaction with environmental stressors. Here, in a pre-adolescent population-based sample, we used polygenic risk scores from a case-control discovery sample of the Psychiatric Genomics Consortium as indicators of genetic vulnerability to schizophrenia. In addition, hair cortisol levels were obtained as a naturalistic quantitative metric of long-term physiological stress. We examined whether cortisol levels moderated the relationship between schizophrenia polygenic risks scores and pre-adolescent brain structure.

**Methods:**

This study was embedded in the Generation R Study, a prospective birth cohort from the Netherlands. Polygenic risk scores for schizophrenia were calculated in children of European ancestry only. P-value thresholds for inclusion of genetic variants in the polygenic risk score varied between P < 0.001 and P < 1. Hair cortisol was collected when the children were approximately 6 years old. At age 9 years, children underwent a magnetic resonance imaging (MRI) procedure to assess volumetric brain measures. After genetic and neuroimaging quality control procedures, the final sample consisted of 522 participants. Linear regression models were conducted to examine the associations between schizophrenia polygenic risk scores, hair cortisol levels, and brain volumes. All analyses were adjusted for age, sex, hair color, hair product use and four genetic principal components.

**Results:**

Schizophrenia polygenic risk scores were not associated with hair cortisol levels (P-value threshold [PT] < 0.001, β = -0.03, 95% confidence interval [CI] -0.11 – 0.05, P = 0.441), cortical grey matter volume (PT < 0.001, β = -0.03, 95% CI -0.11 – 0.05, P = 0.457) or cerebral white matter volume (PT < 0.001, β = -0.04, 95% CI -0.12 – 0.04, P = 0.356). Higher schizophrenia polygenic risk scores were associated with lower total ventricle volume (PT <0.001, β = -0.10, 95% CI -0.19 – -0.02, P = 0.022), including when mutually adjusted for total brain volume. Notably however, hair cortisol exhibited a positive interaction with schizophrenia risk scores in predicting total ventricle volume (PT < 0.001, β = 0.10, 95% CI 0.01 – 0.18, P = 0.027), i.e. higher schizophrenia polygenic risk score and higher hair cortisol levels were associated with increased ventricle volumes. Hair cortisol was not independently associated with total ventricle volume (PT < 0.001, β = -0.05, 95% CI -0.14 – 0.03, P = 0.215).

**Discussion:**

Elevated hair cortisol levels, a biological index of long-term stress, moderated the association between genetic vulnerability to schizophrenia and total cerebral ventricle volume among pre-adolescents. These findings underscore the importance of the interplay between genes and environment in shaping brain development. Using a polygenic risk score for schizophrenia, we provide novel, albeit preliminary, evidence for gene-environment interaction between genetic risk for schizophrenia and environmental stressors in the general pre-adolescent population. This may help to elucidate underlying etiologies and possible early interventions for the psychosis spectrum.

